# Ethical Concerns of Research Ethics Committees in Suicide Research: A Qualitative Study From Pakistan

**DOI:** 10.1192/bjo.2024.207

**Published:** 2024-08-01

**Authors:** Muqaddas Asif, Rakhshi Memon, Ameer B. Khoso, Zaib un Nisa, Nusrat Husain

**Affiliations:** 1 Pakistan Institute of Living and Learning, Karachi, Pakistan; 2 University of Manchester, Manchester, United Kingdom; 3 University College London, London, United Kingdom

## Abstract

**Aims:**

Suicide is a global public health issue that requires sensitive research to inform effective prevention and treatment strategies. Despite the benefits of such research, it is accompanied by significant ethical challenges such as the potential for harm to participants' wellbeing. Various studies have explored the views of researchers in suicide research. This qualitative study aimed to explore the research ethics committee (REC) members’ experiences with suicide-related study applications to ascertain whether there are differences in approaches to dealing with suicide-related study applications.

**Methods:**

We conducted semi-structured interviews with members of RECs (n = 9) from research-intensive universities and ethics committees in Pakistan. We also conducted a discussion group (n = 13) with members of REC from Pakistan, Nigeria and Sri Lanka. The topic guide delved into the opinions of REC members regarding ethical issues that they have come across while reviewing self-harm/suicide-related research proposals, the relevance of these issues with specific study designs, recommendations to resolve these issues, their approach to balancing risk and benefit, and guidance for researchers.

**Results:**

The preliminary findings from thematic analysis revealed five major themes; 1) Ethical challenges, 2) Reasons for application rejection, 3) Areas of improvement, 4) Suggestions for addressing ethical issues, and 5) Researchers' attitudes towards amendments. Challenges in self-harm and suicide research included the sensitivity and stigma surrounding the topic, lack of interest and support, and difficulties in participant recruitment. The application faced rejection from the ethics committees primarily due to methodological errors, lack of procedural clarity, and insufficient understanding of the research procedure. Identified areas for improvement were the need for enhanced methodology and research patterns, as well as a better understanding of the methodological procedure. Recommendations for developing a robust research proposal included training and supervision for intervention studies, the inclusion of comprehensive ethical elements and practical plans in the proposal, and a focus on data protection, confidentiality, risk management, and harm identification. While a significant number positively acknowledged reviewer comments, some researchers opted for in-depth discussions rather than directly addressing the issues.

**Conclusion:**

The study highlights the importance of ethical considerations and emphasises the need to address the lack of robust methodological procedures in self-harm and suicide research. Addressing these challenges and adopting suggested improvements is paramount for advancing ethical and impactful research in this context.

